# Synthesis and Antibacterial Activity of Manganese-Ferrite/Silver Nanocomposite Combined with Two Essential Oils

**DOI:** 10.3390/nano12132137

**Published:** 2022-06-22

**Authors:** Javiera Parada, Marcela Díaz, Edward Hermosilla, Joelis Vera, Gonzalo Tortella, Amedea B. Seabra, Andrés Quiroz, Emilio Hormazábal, Olga Rubilar

**Affiliations:** 1 Chemical Engineering Department, Universidad de La Frontera, Temuco P.O. Box 54-D, Chile; javiera.parada@ufrontera.cl (J.P.); edward.hermosilla@ufrontera.cl (E.H.); gonzalo.tortella@ufrontera.cl (G.T.); 2 Biotechnological Research Center Applied to the Environment (CIBAMA-BIOREN), Universidad de La Frontera, Temuco P.O. Box 54-D, Chile; marcela.diaz@ufrontera.cl (M.D.); j.vera12@ufromail.cl (J.V.); 3 Programa de Doctorado en Ciencias de la Ingeniería, Universidad de La Frontera, Temuco P.O. Box 54-D, Chile; 4 Center for Natural and Human Sciences, Federal University of ABC (UFABC), Santo André 09210-580, Brazil; amedea.seabra@ufabc.edu.br; 5 Departamento de Ciencias Químicas y Recursos Naturales, Universidad de La Frontera, Temuco P.O. Box 54-D, Chile; andres.quiroz@ufrontera.cl (A.Q.); emilio.hormazabal@ufrontera.cl (E.H.)

**Keywords:** manganese-ferrite nanoparticles, silver nanoparticles, nanocomposite, antibacterial, essential oils

## Abstract

The antimicrobial activity of metal nanoparticles obtained by biogenic routes has been extensively reported. However, their combined use with other antimicrobial formulations, such as essential oils, remains scarcely explored. In this work, a manganese-ferrite/silver nanocomposite (MnFe_2_O_4_/Ag-NC) was synthesized in a two-step procedure: first, MnFe_2_O_4_ nanoparticles were produced by a coprecipitation method, followed by *in situ* biogenic reduction of silver ions using *Galega officinalis*. MnFe_2_O_4_/Ag-NC was characterized using transmission electron microscopy (TEM), scanning electron microscopy equipped with an energy dispersive X-ray analyzer (SEM-EDX), and a vibrating sample magnetometer (VSM-SQUID). The antibacterial activity if MnFe_2_O_4_/Ag-NC was evaluated against *Pseudomonas syringae* by determining its minimum inhibitory concentration (MIC) in the presence of two essential oils: eucalyptus oil (EO) and garlic oil (GO). The fractional inhibitory concentration (FIC) was also calculated to determine the interaction between MnFe_2_O_4_/Ag-NC and each oil. The MIC of MnFe_2_O_4_/Ag-NC was eightfold reduced with the two essential oils (from 20 to 2.5 µg mL^−1^). However, the interaction with EO was synergistic (FIC: 0.5), whereas the interaction with GO was additive (FIC: 0.75). Additionally, a time-kill curve analysis was performed, wherein the MIC of the combination of MnFe_2_O_4_/Ag-NC and EO provoked a rapid bactericidal effect, corroborating a strong synergism. These findings suggest that by combining MnFe_2_O_4_/Ag-NC with essential oils, the necessary ratio of the nanocomposite to control phytopathogens can be reduced, thus minimizing the environmental release of silver.

## 1. Introduction

The development of efficient and ecofriendly technologies for agriculture management has been one of the most significant challenges for the scientific community. In this context, nanotechnology has emerged as a relevant discipline in the last decade, and a considerable variety of nanoproducts has been produced with the aim of mitigating the effects of the overuse of agrochemicals (e.g., nanofertilizers, nanopesticides, or nanocomposites) [1,2]. The enhanced physicochemical properties provided by the high surface area-to-volume ratio of metal nanoparticles has led to their use as antibacterial agents against many pathogens. In this regard, silver nanoparticles (AgNPs) are one of the most commonly used antibacterial agents due to their well-known enhanced antimicrobial properties and thermal stability compared to other metals [3,4]. The antimicrobial effect of AgNPs has been demonstrated to be more potent when compared to that of silver ions [5]. One of the main shortcomings of using AgNPs is their reported ecotoxicity once released into the environment when used in high concentrations, even imposing a potential risk of human exposure [6,7]. Formulations of magnetic nanocomposites based on magnetite or manganese-ferrite nanoparticles (MnFe_2_O_4_-NPs) have been proposed as carriers of AgNPs that allow for the reuse of silver, which is possible through an enhanced separation process with the use of a magnetic field. This is a valuable strategy for recycling magnetic nanoparticles and silver-magnetic nanocomposites from an aqueous medium that can minimize the environmental release of silver [8,9]. Other advantages of using MnFe_2_O_4_-NPs include their stability in air and high magnetic saturation relative to other magnetic materials [10]. In this context, nanocomposites based on MnFe_2_O_4_-NPs and silver have been mainly studied for environmental applications due to their ability to decompose dyes in water resources, as well as to adsorb contaminants [11,12,13]. However, very few studies have addressed their reusability or antibacterial effectiveness [14]. A two-stage chemical procedure is the most extensively reported method for elaborating nanocomposites based on MnFe_2_O_4_-NPs and AgNPs. First, MnFe_2_O_4_-NPs are synthesized by different routes (e.g., coprecipitation techniques, hydrothermal methods, or other techniques). Secondly, the obtained nanoparticles are functionalized with ligand groups that attach AgNPs onto the surface of MnFe_2_O_4_-NPs. The adhesion force of AgNPs and the expensiveness of the chemical reagents used during the process are among the issues associated with this method [15]. To overcome these issues, green routes have been recently proposed as an alternative to the use of chemically mediated methods for the synthesis of MnFe_2_O_4_-NPs [16]. In this regard, *Galega officinalis*, a plant belonging to the Fabaceae family, is a potential candidate that has been demonstrated to be effective in the synthesis of AgNPs, which can be attributed to its high content of flavonoids and polyphenols [17,18]. 

On the other hand, the antimicrobial activity of some essential oils or their nanoformulations against a variety of microorganisms has long been studied [19,20]. In this sense, synergistic or additive interaction has been reported for some essential oils evaluated in combination with other antibiotics [21,22], and currently, their combination with nanoparticles or nanocomposites has attracted considerable interest due to the promising results obtained with respect to the control of pathogenic microorganisms [23,24,25]. Moreover, it is worth noting that essential oils derived from plants are considered safer for the environment than commercial pesticides, making them ideal for agronomic applications [26,27]. In the present study, we performed a plant-mediated synthesis of a magnetic manganese-ferrite/silver nanocomposite (MnFe_2_O_4_/Ag-NC), and its antimicrobial activity was evaluated on the plant phytopathogenic bacteria *Pseudomonas syringae* as a test model. Additionally, to reduce the amount of MnFe_2_O_4_/Ag-NC necessary to inhibit bacterial growth, its combined effect with two essential oils (eucalyptus and garlic) was evaluated.

## 2. Materials and Methods

### 2.1. Materials

Ferreous chloride (FeCl_2_), manganese dichloride (MnCl_2_), sodium hydroxide (NaOH), and silver nitrate (AgNO_3_) of analytical grade were purchased from Merck S.A. Garlic essential oil was purchased from Sigma Aldrich (St. Louis, MO, USA), and eucalyptus essential oil was extracted using a steam distillation method. The experimental bacterial strain (*Pseudomonas* *syringae* Ps-Nt-2016) was obtained from the Chilean Culture Collection of Universidad de La Frontera (CCCT-UFRO). This bacterial strain was cultured according to standard guidelines (CLSI 2012). Antimicrobial assays were performed in Mueller–Hinton medium (broth and agar). All assays were performed in Milli-Q water.

### 2.2. Characterization of the Essential Oils

Garlic and eucalyptus essential oils were chemically characterized (Table 1) using a gas chromatograph (GC) (model Focus, Thermo Electron, Waltham, MA, USA) coupled with a mass spectrometer (model DSQ, Thermo Electron, Waltham, MA, USA) equipped with a capillary column (HP-5, SGE HP-5, 30 m × 0.25 mm × 0.25 μm, SGE, Ringwood, VIC, Australia). Helium was used as carrier gas with a flow rate of 1 mL/min. MS acquisition was performed in the mass range of 35 to 500 *m*/*z*, followed by ionization by electron impact ionization at 70 eV. The injector and transfer line were fixed at 250 °C and 200 °C, respectively. The GC oven temperature started at 40 °C for 3 min, then increased to 250 °C at a rate of 5 °C/min. This analytical method has been previously validated [28]. Afterward, monoterpenes were identified using a series of alkanes (C_9_–C_18_) by comparing the experimental and theoretical Kovats indices (KI) of each compound according to the National Institute of Standards and Technology (NIST) library. This identification was corroborated by a comparison of the mass spectra with a library database (NIST ver. 2.0, Gaithersburg, MD, USA).

### 2.3. Synthesis of Manganese-Ferrite/Silver Nanocomposite (MnFe_2_O_4_/Ag-NC)

MnFe_2_O_4_-NPs were synthesized using a method of coprecipitation. In detail, FeCl_2_ 4 M and MnCl_2_ 2 M (ratio 2:1) were mixed under continuous agitation. A volume of 17 mL of NaOH 7M was added to obtain a basic pH in the reaction. The process was performed in a thermostatic bath at ebullition for 2 h. The obtained MnFe_2_O_4_-NPs were washed using a neodymium magnet and lyophilized. As a second step, the MnFe_2_O_4_-NPs were functionalized with AgNPs using an extract of *Galega officinalis* according to the method described by Manosalva et al. [18]. The extract was obtained by the ebullition of 10 g of leaves in 100 mL of deionized water. This solution was filtered and diluted to 10% *v*/*v* to obtain 1 L; then, 400 mg of MnFe_2_O_4_-NPs was added and 100 mL of AgNO_3_ 120 mM was added dropwise. This mixture was maintained under agitation at 150 rpm for 3 h. Finally, the obtained MnFe_2_O_4_/Ag-NC was washed with water three times and separated with a neodymium magnet to be dried at 60 °C for 12 h. The entire process of synthesis and characterization of the MnFe_2_O_4_/Ag-NC was repeated three times to assure its reproducibility.

### 2.4. Characterization of the MnFe_2_O_4_/Ag-NC

The particle size and shape of the MnFe_2_O_4_-NPs and MnFe_2_O_4_/Ag-NC were characterized by transmission electron microscopy (TEM) using a JEM 2100 B6 microscope (JEOL, Akishima, TYO, Japan) operating at a resolution of 0.25 nm point-to-point and 200 kV of acceleration voltage. Surface morphology and elemental analyses were performed using a HITACHI SU3500 scanning electron microscope (Japan) fitted with an electron-dispersive X-ray spectrometer (SEM/EDX) (Hitachi, TYO, Japan) at 15.0 kV. Magnetic properties were measured by a vibrating sample magnetometer (VSM-SQUID, Quantum Design, Inc., San Diego, CA, USA) at 300 K as a function of the applied magnetic field of −3 to +3 Tesla with powder samples. X-ray diffraction (XRD) analysis of MnFe_2_O_4_-NPs and MnFe_2_O_4_/Ag-NC was performed using a STADI-P diffractometer (Stoe^®^, Darmstadt, Germany) operating at room temperature, 50 kV, and 40 mA and using MoKα1 (λ = 0.7093 Å) radiation. Data were recorded in the 2θ range of 5.0° to 45°, with step sizes of 0.015° and a counting time of 100 s for every 0.785°. Qualitative and semi-quantitative phase analyses of XRD data were conducted utilizing X’Pert HighScore software (ver. 2.0) with a version of the PDF-2.

### 2.5. Antimicrobial Activity: Checkerboard Assay

The antimicrobial activity of MnFe_2_O_4_/Ag-NC against *Pseudomonas syringae* Ps-Nt-2016 was evaluated in the presence of two essential oils: eucalyptus oil (EO) and garlic oil (GO). The minimum inhibitory concentration (MIC) of the MnFe_2_O_4_/Ag-NC, either alone or combined with the essential oils, was determined through the measured turbidity (optical density at 600 nm; OD_600_). Additionally, in order to detect potential synergism or additive effects between the two components, the fractional inhibitory concentration (FIC) was also determined by the checkerboard dilution test described by Hsieh et al. [29]. First, *P. syringae* was cultured in sterile Mueller–Hinton broth (MHB) at 28 °C to reach the value of OD_600_ equivalent to ~1 to 2 × 10^6^ CFU mL^−1^. Then, 20 µL culture aliquots were transferred to a 96-well plate to perform a checkerboard assay. The treatments on the 96 well-plate consisted of twofold serial dilutions of MnFe_2_O_4_/Ag-NC (2.5–160 µg mL^−1^) combined with the essential oils (EO: 0.25–16 mg mL^−1^; GO: 0.04–2.4 mg mL^−1^). These concentrations were selected according to previous experiments performed against *P. syringae* Ps-Nt-2016. The MnFe_2_O_4_-NPs were not considered in this assay because according to a preliminary experiment, they did not exhibit antimicrobial activity against *P. syringae*. All treatments were carried out in triplicate, and positive and negative controls were included. After a static 24 h incubation of the plate, the OD_600_ was measured with an Epoch Spectrophotometer system (BioTek Instrument Inc., Winooski, VT, USA). OD_600_ ≥ 0.07 was considered as bacterial growth, ≥0.01 and <0.07 as inhibitory and <0.01 as bactericide. These selection criteria were chosen according to the previously determined growth curve of *P. syringae* Ps-Nt-2016s. The MIC was defined as the lowest concentration to inhibit bacterial growth. An aliquot from wells with OD_600_ < 0.01 was inoculated in Mueller–Hinton agar (MHA) to test the bactericidal effect at 28 °C for 24 h, as well as the absence of colonies. The FIC value was determined according to Equation (1):

(1) $$\frac{A}{{\mathrm{MIC}}_{A}}+\frac{B}{{\mathrm{MIC}}_{B}}={\mathrm{FIC}}_{A}+{\mathrm{FIC}}_{B}=\mathrm{FIC}$$

where A and B correspond to the MIC of the essential oils and MnFe_2_O_4_/Ag-NC used in combination. MIC_A_ and MIC_B_ correspond to the MIC of each component used individually. The FIC value was used to categorize the interaction as synergistic (≤0.5), additive (0.5–1), or indifferent (1–4).

### 2.6. Time-Kill Curve Assay

Time-kill curve assays were performed to monitor the effect on the growth and death of *P. syringae*. The concentrations evaluated were the MIC of each agent alone according to the checkerboard assay (MnFe_2_O_4_/Ag-NC: 20 µg mL^−1^; EO: 4 mg mL^−1^; GO: 0.14 mg mL^−1^) and the respective combinations. These concentrations were chosen to corroborate the synergism or additivity evidenced by the FIC value. In brief, a bacterial suspension of ~1 to 2 × 10^6^ CFU mL^−1^ in MHB was mixed with the treatment in a ratio of 1:1. Aliquots of all treatments were sampled at 0, 2, 3, 5, 7, 12, and 24 h and serially diluted to be cultured on MHA. Thus, the obtained colony-forming units (CFU) were counted to construct the kill curves. 

## 3. Results and Discussion

### 3.1. Characterization of MnFe_2_O_4_ and MnFe_2_O_4_/Ag-NC

**Figure 1:**
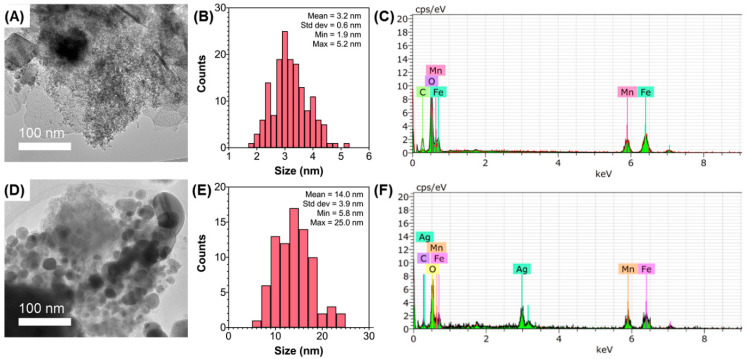
TEM images, histograms of size distribution, and EDX spectra of the powdered samples of MnFe_2_O_4_-NPs (**A**–**C**) and MnFe_2_O_4_/Ag-NC (**D**–**F**).

According to the results of the characterization and TEM images shown in Figure 1, the average size of the MnFe_2_O_4_-NPs and the MnFe_2_O_4_/Ag-NC was ~3 nm and ~14 nm, respectively. The coprecipitation method also resulted in an increase in the size of MnFe_2_O_4_-Ag hybrid nanoparticles, as reported by Nha et al. [10]. However, the in situ biogenic reduction with *G. officinalis* used in this study may have added other elements behaving as stabilizing agents, leading to a larger size of the MnFe_2_O_4_/Ag-NC. The results of energy-dispersive analysis of X-ray spectroscopy (EDX) obtained by SEM-EDX confirmed the presence of manganese and iron in MnFe_2_O_4_-NPs, as well as the presence of manganese, iron, and silver on MnFe_2_O_4_/Ag-NC (shown in Figure 1C,F). The observed oxygen peaks were attributed to the presence of oxides and the carbon peaks corresponding to the carbon tape used to prepare the samples. In this sense, the corresponding elemental mapping (shown in Supplementary Figure S1) evidenced that manganese and iron were evenly spaced on both samples. Similarly, silver was homogenously distributed on the MnFe_2_O_4_/Ag-NC, suggesting an effective functionalization of MnFe_2_O_4_-NPs. 

**Figure 2:**
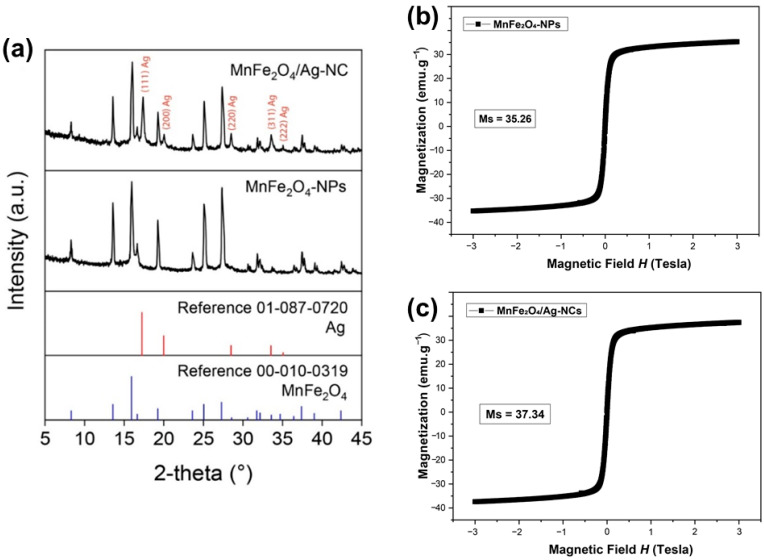
XRD patterns (**a**) and Hysteresis loop (**b**,**c**) of MnFe_2_O_4_ and MnFe_2_O_4_/Ag-NC.

The formation of the spinel MnFe_2_O_4_ was corroborated by XRD analysis (Figure 2a). Thus, the sample of MnFe_2_O_4_-NPs was consistent with the MnFe_2_O_4_ (manganese iron oxide) structure, which matched with the reference pattern ICDD 00-010-0319. Qualitative phase analysis of the XRD spectra of MnFe_2_O_4_/Ag-NC (performed with X’Pert HighScore software) indicated that MnFe_2_O_4_ is the most relevant phase in the sample with the presence of an Ag^0^ phase (ICDD 01-087-0720). Semi-quantitative phase analysis of MnFe_2_O_4_/Ag-NC resulted in MnFe_2_O_4_ (90% *w/w*) and Ag^0^ (10% *w/w*). These results suggest that in the second step of MnFe_2_O_4_/Ag-NC synthesis, the *G. officinalis* extract reduced Ag^+^ ions of AgNO_3_ to Ag^0^, which was deposited on the MnFe_2_O_4_-NPs surface. The saturation magnetization of the synthesized MnFe_2_O_4_-NPs was 35.26 emu g^−1^ (Figure 2b) and 37.34 emu g^−1^ for MnFe_2_O_4_/Ag-NC (Figure 2c). This demonstrates that the functionalization with silver provoked minimal changes in the magnetic properties of the MnFe_2_O_4_-NPs. Values from 30 to 69 emu g^−1^ have been reported for MnFe_2_O_4_-NPs [30]. We observed superparamagnetic behavior in both samples. No coercivity or remanence was observed on hysteresis loops of MnFe_2_O_4_-NPs and MnFe_2_O_4_/Ag-NC.

### 3.2. Antimicrobial Activity of MnFe_2_O_4_/Ag-NC Combined with Essential Oils

**Figure 3:**
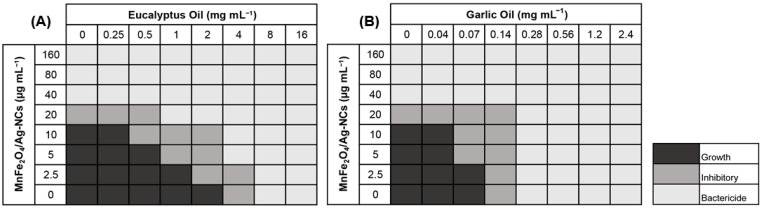
Checkerboard assay performed with MnFe_2_O_4_/Ag-NC combined with eucalyptus oil (**A**) and garlic oil (**B**) against *Pseudomonas syringae*.

According to the results obtained from the checkerboard assay (shown in Figure 3), the MnFe_2_O_4_/Ag-NC synthesized in this study inhibited the growth of *P. syringae*, which improved in combination with the two essential oils: eucalyptus oil (EO) and garlic oil (GO). These oils were selected due to their known antibacterial activity against a large diversity of pathogenic bacterial strains, either alone or combined with other antibiotics [20,31,32]. The MIC of MnFe_2_O_4_/Ag-NC used individually was 20 µg mL^−1^, but combined with 2 mg mL^−1^ of EO or 0.14 mg mL^−1^ of GO, this value was reduced to 2.5 µg mL^−1^ (equivalent to 0.25 µg mL^−1^ of silver, considering that 10% *w/w* of the MnFe_2_O_4_/Ag-NC is composed of silver). 

On the other hand, the MIC values of the EO and GO individually were 4 and 0.14 mg mL^−1^, respectively, but these were reduced to 0.25 and 0.04 mg mL^−1^ in combination with 20 µg mL^−1^ of MnFe_2_O_4_/Ag-NC. It is important to mention that depending on the antimicrobial effect, essential oils can be classified as strong for MIC values lower than 0.5 mg mL^−1^, as moderate for MIC values between 0.5 and 1.5 mg mL^−1^, and as weak for MIC values higher than 1.6 mg mL^−1^ [33]. This antecedent is relevant because EO alone behaved as a weak antimicrobial against *P. syringae* (MIC: 4 mg mL^−1^) and strong when combined with 10 or 20 µg mL^−1^ of MnFe_2_O_4_/Ag-NC (MIC: <0.5 mg mL^−1^), whereas GO behaved as a strong antimicrobial individually and even more strongly in the presence of MnFe_2_O_4_/Ag-NC. 

To determine the type of interaction involved in these two combinations, the FIC was calculated on those wells where the bacterial growth was inhibited [29]. The lowest FIC for both combinations (Table 2) demonstrated that the interaction between MnFe_2_O_4_/Ag-NC and EO was synergistic (FIC: 0.5). This synergism index was obtained with 5 µg mL^−1^ of MnFe_2_O_4_/Ag-NC (equivalent to 0.5 µg mL^−1^ of silver) and 1 mg mL^−1^ of EO. These concentrations were lower than those obtained in a similar study performed by Asghar Heydari et al. [32], who reported that a combination of commercial AgNPs (1.5 µg mL^−1^) and EO (6.25 mg mL^−1^) also exerted a synergistic effect against *Escherichia coli*, *Staphylococcus aureus*, *Salmonella enterica*, and *Bacillus subtilis*. The interaction of MnFe_2_O_4_/Ag-NC with GO obtained with 5 µg mL^−1^ of MnFe_2_O_4_/Ag-NC and 0.07 mg mL^−1^ of GO was additive (FIC: 0.75). Although this effect was not synergistic, it should be noted that the GO MIC was reduced from 0.14 to 0.04 mg mL^−1^ in the presence of MnFe_2_O_4_/Ag-NC, and this amount is lower than that obtained by Zabihi et al., who reported an MIC of 0.3 mg mL^−1^ of GO against *E. coli* [34].

**Figure 4:**
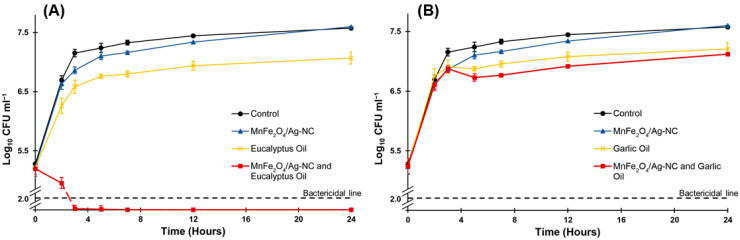
Time-kill curve for *Pseudomonas syringae* treated with MnFe_2_O_4_/Ag-NC (20 µg mL^−1^) combined with eucalyptus oil (4 mg mL^−1^) (**A**) and MnFe_2_O_4_/Ag-NC (20 µg mL^−1^) combined with garlic oil (0.14 mg mL^−1^) (**B**). Each agent was also evaluated individually at the same concentrations. Error bars indicate standard deviation.

In terms of the silver concentration, studies have reported that inhibitory doses higher than 10 µg mL^−1^ of different biogenic AgNPs inhibit *P. syringae*, either alone or combined with other antibiotics [35,36,37,38]. Thus, by using MnFe_2_O_4_/Ag-NC, we reported a further reduced concentration of silver acting efficiently against *P. syringae* when combined with essential oils. Moreover, the results of the time-kill curve assay corroborated the synergism between MnFe_2_O_4_/Ag-NC and EO (Figure 4A). In particular, the number of CFUs was reduced by more than two logarithmic units following 3 h of exposure to the combination of MnFe_2_O_4_/Ag-NC (MIC: 20 µg mL^−1^) and EO (MIC: 4 mg mL^−1^), implying a bactericidal effect. However, the effect with each agent used individually was merely inhibitory. The additive effect of MnFe_2_O_4_/Ag-NC combined with GO was also confirmed (Figure 4B). However, in contrast to the results obtained with EO, only an inhibitory effect was observed, which can be explained by the sum of the effect of each agent used individually. However, it is important to note that a bactericidal effect of this combination occurs with the use of a higher amount of GO. 

The additive or synergistic effect obtained between the MnFe_2_O_4_/Ag-NC and the essential oils could be related to the fact that both combinations collaborate through similar and different antibacterial mechanisms (i.e., targeting different cell parts). On the one hand, many mechanisms have been associated with AgNPs (contained in the MnFe_2_O_4_/Ag-NC). However, their binding capacity with sulfhydryl and phosphate groups present on the cell membrane was confirmed, which leads to membrane and enzymatic dysfunction and bacterial lysis [5,18]. On the other hand, the hydrophobicity of the essential oils facilitates their attachment to the thick lipopolysaccharide membrane of Gram-negative bacteria, which could explain the observed susceptibility of *P. syringae*. Nevertheless, eucalyptol, one of the main constituents of EO (Table 1) has been demonstrated to cause intracellular damage instead of membrane damage. In contrast, the organosulfur compounds present in GO form disulfide bonds with sulfhydryl groups, which inactivates enzymes, leading to bacterial death [39,40,41]. Thus, recognizing that multiple mechanisms can be involved in these interactions, we hypothesize that MnFe_2_O_4_/Ag-NC provokes membrane permeability damage, whereas EO produces intracellular alterations, leading to a more potent effect and synergism. The additive effect of MnFe_2_O_4_/Ag-NC with GO can be associated with both compounds working on the same target. However, further mechanistic studies are necessary to complement the use of this nanocomposite with essential oils.

## 4. Conclusions

In this study, we obtained a nanocomposite (MnFe_2_O_4_/Ag-NC), and the second step of the synthesis process (reduction of silver ions) was successfully performed via a biogenic route using *Galega officinalis.* This evidenced the possibility of reducing the costs of the reagents commonly used in that step (e.g., polyvinylpyrrolidone, 3-aminopropyltriethoxysilane, and polyaniline), both in terms of expense and environmental impact. More importantly, the amount of the MnFe_2_O_4_/Ag-NC necessary to inhibit *P. syringae* was reduced by 8-fold when combined with eucalyptus and garlic essential oils (both selected due to their known antibacterial activity). Herein, eucalyptus oil exhibited a potent synergistic interaction with MnFe_2_O_4_/Ag-NC, whereas the interaction with garlic oil was additive. The aforementioned support the feasibility of using the MnFe_2_O_4_/Ag-NC combined with eucalyptus oil as an alternative to conventional pesticides. More studies should be conducted in order to improve their efficacy, for instance, by obtaining a stable nanoformulation based on both agents, assuming the high volatility of eucalyptus oil. Furthermore, considering its magnetic nature, the reusability of MnFe_2_O_4_/Ag-NC should be evaluated to further minimize the environmental impact of silver.

These findings suggest the combination of MnFe_2_O_4_/Ag-NC with eucalyptus oil as a novel and helpful alternative to reduce the overuse of conventional pesticides. Considering that this study represents a first approach, more bacterial strains need to be tested in future studies in order to obtain a wider range of applications for this combination. Additionally, studies on the potential toxicity of this combination in different organisms could provide valuable information that could be used to prevent non-target effects in the environment.

## Figures and Tables

**Table 1:** Chemical composition of eucalyptus and garlic essential oils according to GC-MS analysis. (RT = retention time; KI = Kovats index; KI exp = experimental Kovats index; KI lib = Kovats index database).

Essential Oil	RT (min)	Compound	Area (%)	KI Exp.	KI Lib.
**Eucalyptus**	9.76	a-Pinene	20.9	936	929
	10.83	β-Pinene	3.4	970	979
	11.48	β-Myrcene	1.4	990	991
	12.42	Eucalyptol	46.0	1021	1032
	16.76	Terpinen-4-ol	1.3	1166	1177
	16.99	Verbenyl ethyl ether	3.7	1173	1186
	17.11	α-Terpineol	0.2	1177	1189
	21.14	exo-Hydroxycineole acetate	0.5	1321	1344
	21.47	α-Terpenyl acetate	5.9	1334	1350
	22.42	Isoledene	0.5	1370	1375
	22.50	Copaene	0.3	1373	1376
	23.34	β-Maatiene	1.0	1404	1405
	24.08	Aromadendrene	4.9	1435	1440
	24.54	9-epi-β-Caryophyllene	1.8	1453	1466
	25.40	Ledene	0.8	1487	1493
	26.85	Epiglobulol	1.2	1549	1580
	27.41	Globulol	4.1	1572	1585
	27.56	Viridiflorol	1.7	1579	1591
	27.84	Rosifoliol	0.5	1590	1649
**Garlic**	8.89	Diallyl sulfide	7.3	905	861
	10.69	Methyl 2-propenyl disulfide	13.7	966	920
	11.75	1,2-Dithiole	19.7	997	952
	12.53	Dimethyl trisulfide	1.4	1025	970
	16.46	Diallyl disulfide	15.5	1156	1081
	17.02	1(E)-1-Propen-1-yl 2-propenyl disulfide	0.8	1174	1103
	18.12	Allyl methyl trisulfide	4.4	1211	1142
	19.56	3-Vinyl-1,2-dithiacyclohex-4-ene	0.2	1264	1198
	19.63	1,2,3-Trithia-4-cyclohexene	4.1	1266	1202
	24.53	5-Methyl-1,2,3,4-tetrathiane	14.1	1446	1364

**Table 2:** Minimum inhibitory concentration (MIC) and fractional inhibitory concentration (FIC) of MnFe_2_O_4_/Ag-NC combined with essential oils against *Pseudomonas syringae.*

Combination	MIC		FIC	Interaction Type
	Alone	Combined		
MnFe_2_O_4_/Ag-NC and Eucalyptus oil	20 µg mL^−1^	5 µg mL^−1^	0.5	Synergistic
	4 mg mL^−1^	1 mg mL^−1^		
MnFe_2_O_4_/Ag-NC and Garlic oil	20 µg mL^−1^	5 µg mL^−1^	0.75	Additive
	0.14 mg mL^−1^	0.07 mg mL^−1^		

## Data Availability

Not applicable.

## Supplementary Materials

The following supporting information can be downloaded at: https://www.mdpi.com/article/10.3390/nano12132137/s1, Figure S1: EDS elemental mapping for MnFe_2_O_4_ (A) and MnFe_2_O_4_/Ag-NC (B).

## References

[1] Guha T., Gopal G., Kundu R., Mukherjee A. (2020). Nanocomposites for Delivering Agrochemicals: A Comprehensive Review. J. Agric. Food Chem..

[2] Nehra M., Dilbaghi N., Marrazza G., Kaushik A., Sonne C., Kim K.H., Kumar S. (2021). Emerging nanobiotechnology in agriculture for the management of pesticide residues. J. Hazard. Mater..

[3] Franci G., Falanga A., Galdiero S., Palomba L., Rai M., Morelli G., Galdiero M. (2015). Silver Nanoparticles as Potential Antibacterial Agents. Molecules.

[4] Bedlovičová Z., Salayová A. (2018). Green-Synthesized Silver Nanoparticles and Their Potential for Antibacterial Applications. Bacterial Pathogenesis and Antibacterial Control.

[5] Yan X., He B., Liu L., Qu G., Shi J., Hu L., Jiang G. (2018). Antibacterial mechanism of silver nanoparticles in *Pseudomonas aeruginosa*: Proteomics approach. Metallomics.

[6] Parada J., Rubilar O., Diez M.C., Cea M., Sant’Ana da Silva A., Rodríguez-Rodríguez C.E., Tortella G.R. (2019). Combined pollution of copper nanoparticles and atrazine in soil: Effects on dissipation of the pesticide and on microbiological community profiles. J. Hazard. Mater..

[7] Tortella G.R., Rubilar O., Durán N., Diez M.C., Martínez M., Parada J., Seabra A.B. (2020). Silver nanoparticles: Toxicity in model organisms as an overview of its hazard for human health and the environment. J. Hazard. Mater..

[8] Joshi M.K., Pant H.R., Liao N., Kim J.H., Kim H.J., Park C.H., Kim C.S. (2015). In-situ deposition of silver-iron oxide nanoparticles on the surface of fly ash for water purification. J. Colloid Interface Sci..

[9] Fang W., Zheng Q., Fang Y., Huang H. (2019). Facile synthesis of silver-decorated magnetic nanospheres used as effective and recyclable antibacterial agents. Curr. Appl. Phys..

[10] Nha T.T.N., Nam P.H., Phuc N.X., Nguyen V.Q., Nam N.H., Manh D.H., Tam L.T., Linh N.T.N., Khanh B.T.V., Lu L.T. (2021). Sensitive MnFe_2_O_4_–Ag hybrid nanoparticles with photothermal and magnetothermal properties for hyperthermia applications. RSC Adv..

[11] Amir M., Kurtan U., Baykal A., Sözeri H. (2016). MnFe_2_O_4_@PANI@Ag Heterogeneous Nanocatalyst for Degradation of Industrial Aqueous Organic Pollutants. J. Mater. Sci. Technol..

[12] Huy L.T., Tam L.T., Phan V.N., Trung T., Tung L.M., Thanh D.T.N., Hoa N.Q., Vinh L.K., Ngo D.T., Mølhave K. (2016). Effect of synthesis parameters on the structure and magnetic properties of magnetic manganese ferrite/silver composite nanoparticles synthesized by wet chemistry method. J. Nanosci. Nanotechnol..

[13] Desai H.B., Hathiya L.J., Joshi H.H., Tanna A.R. (2020). Synthesis and characterization of photocatalytic MnFe_2_O_4_ nanoparticles. Mater. Today Proc..

[14] He Q., Liu J., Liang J., Huang C., Li W. (2014). Synthesis and Antibacterial Activity of Magnetic MnFe_2_O_4_/Ag Composite Particles. Nanosci. Nanotechnol. Lett..

[15] Huy L.T., Tam L.T., Van Son T., Cuong N.D., Nam M.H., Vinh L.K., Huy T.Q., Ngo D.T., Phan V.N., Le A.T. (2017). Photochemical Decoration of Silver Nanocrystals on Magnetic MnFe_2_O_4_ Nanoparticles and Their Applications in Antibacterial Agents and SERS-Based Detection. J. Electron. Mater..

[16] Muthukumar H., Palanirajan S.K., Shanmugam M.K., Gummadi S.N. (2020). Plant extract mediated synthesis enhanced the functional properties of silver ferrite nanoparticles over chemical mediated synthesis. Biotechnol. Rep..

[17] Luka C., Adoga G., Istifanus G. (2017). Phytochemical Studies of Different Fractions of Galega officinalis Extract and Their Effects on Some Biochemical Parameters in Alloxan-Induced Diabetic Rats. Eur. J. Med. Plants.

[18] Manosalva N., Tortella G., Cristina Diez M., Schalchli H., Seabra A.B., Durán N., Rubilar O. (2019). Green synthesis of silver nanoparticles: Effect of synthesis reaction parameters on antimicrobial activity. World J. Microbiol. Biotechnol..

[19] Sugumar S., Ghosh V., Nirmala M.J., Mukherjee A., Chandrasekaran N. (2014). Ultrasonic emulsification of eucalyptus oil nanoemulsion: Antibacterial activity against *Staphylococcus aureus* and wound healing activity in Wistar rats. Ultrason. Sonochem..

[20] Jin Z., Li L., Zheng Y., An P. (2020). Inhibition of *Bacillus cereus* by garlic (*Allium sativum*) essential oil during manufacture of white sufu, a traditional Chinese fermented soybean curd. LWT.

[21] Langeveld W.T., Veldhuizen E.J.A., Burt S.A. (2014). Synergy between essential oil components and antibiotics: A review. Crit. Rev. Microbiol..

[22] Duarte A., de Menezes I., Bezerra Morais Braga M., Leite N., Barros L., Waczuk E., Pessoa da Silva M., Boligon A., Teixeira Rocha J., Souza D. (2016). Antimicrobial Activity and Modulatory Effect of Essential Oil from the Leaf of *Rhaphiodon echinus* (Nees & Mart) Schauer on Some Antimicrobial Drugs. Molecules.

[23] Chi H., Song S., Luo M., Zhang C., Li W., Li L., Qin Y. (2019). Effect of PLA nanocomposite films containing bergamot essential oil, TiO_2_ nanoparticles, and Ag nanoparticles on shelf life of mangoes. Sci. Hortic..

[24] Basavegowda N., Patra J.K., Baek K.-H. (2020). Essential Oils and Mono/bi/tri-Metallic Nanocomposites as Alternative Sources of Antimicrobial Agents to Combat Multidrug-Resistant Pathogenic Microorganisms: An Overview. Molecules.

[25] Nair A., Mallya R., Suvarna V., Khan T.A., Momin M., Omri A. (2022). Nanoparticles—Attractive Carriers of Antimicrobial Essential Oils. Antibiotics.

[26] Sharififard M., Safdari F., Siahpoush A., Kassiri H. (2016). Evaluation of Some Plant Essential Oils against the Brown-Banded Cockroach, *Supella longipalpa* (Blattaria: Ectobiidae): A Mechanical Vector of Human Pathogens. J. Arthropod-Borne Dis..

[27] Kah M., Kookana R.S., Gogos A., Bucheli T.D. (2018). A critical evaluation of nanopesticides and nanofertilizers against their conventional analogues. Nat. Nanotechnol..

[28] Tampe J., Espinoza J., Chacón-Fuentes M., Quiroz A., Rubilar M. (2020). Evaluation of *Drimys winteri* (Canelo) Essential Oil as Insecticide against *Acanthoscelides obtectus* (Coleoptera: Bruchidae) and *Aegorhinus superciliosus* (Coleoptera: Curculionidae). Insects.

[29] Hsieh M.H., Yu C.M., Yu V.L., Chow J.W. (1993). Synergy assessed by checkerboard a critical analysis. Diagn. Microbiol. Infect. Dis..

[30] Vernekar A.A., Das T., Ghosh S., Mugesh G. (2016). A Remarkably Efficient MnFe_2_O_4_ -based Oxidase Nanozyme. Chem.-Asian J..

[31] Pereira V., Dias C., Vasconcelos M.C., Rosa E., Saavedra M.J. (2014). Antibacterial activity and synergistic effects between *Eucalyptus globulus* leaf residues (essential oils and extracts) and antibiotics against several isolates of respiratory tract infections (*Pseudomonas aeruginosa*). Ind. Crops Prod..

[32] Asghar Heydari M., Mobini M., Salehi M. (2017). The Synergic Activity of Eucalyptus Leaf Oil and Silver Nanoparticles Against Some Pathogenic Bacteria. Arch. Pediatr. Infect. Dis..

[33] Aligiannis N., Kalpoutzakis E., Mitaku S., Chinou I.B. (2001). Composition and Antimicrobial Activity of the Essential Oils of Two Origanum Species. J. Agric. Food Chem..

[34] Zabihi A., Akhondzadeh Basti A., Amoabediny G., Khanjari A., Tavakkoly Bazzaz J., Mohammadkhan F., Hajjar Bargh A., Vanaki E. (2017). Physicochemical characteristics of nanoliposome garlic (*Allium sativum* L.) Essential Oil and its antibacterial effect on *Escherichia coli* O157:H. J. Food Qual. Hazards Control..

[35] Nikparast Y., Saliani M. (2018). Synergistic Effect between Phyto-Syntesized Silver Nanoparticles and Ciprofloxacin Antibiotic on some Pathogenic Bacterial Strains. J. Med. Bacteriol..

[36] Saratale R.G., Benelli G., Kumar G., Kim D.S., Saratale G.D. (2018). Bio-fabrication of silver nanoparticles using the leaf extract of an ancient herbal medicine, dandelion (*Taraxacum officinale*), evaluation of their antioxidant, anticancer potential, and antimicrobial activity against phytopathogens. Environ. Sci. Pollut. Res..

[37] Gogoi B., Kumar R., Upadhyay J., Borah D. (2020). Facile biogenic synthesis of silver nanoparticles (AgNPs) by *Citrus grandis* (L.) Osbeck fruit extract with excellent antimicrobial potential against plant pathogens. SN Appl. Sci..

[38] Shahryari F., Rabiei Z., Sadighian S. (2020). Antibacterial activity of synthesized silver nanoparticles by sumac aqueous extract and silver-chitosan nanocomposite against *Pseudomonas syringae* pv. syringae. J. Plant Pathol..

[39] He Y., Sang S., Tang H., Ou C. (2022). In vitro mechanism of antibacterial activity of eucalyptus essential oil against specific spoilage organisms in aquatic products. J. Food Process. Preserv..

[40] Mączka W., Duda-Madej A., Górny A., Grabarczyk M., Wińska K. (2021). Can eucalyptol replace antibiotics?. Molecules.

[41] Bhatwalkar S.B., Mondal R., Babu S., Krishna N. (2021). Antibacterial Properties of Organosulfur Compounds of Garlic (*Allium sativum*). Front. Microbiol..

