# Supplementary material for: Synthesis and Antibacterial Activity of Manganese-Ferrite/Silver Nanocomposite Combined with Two Essential Oils
Source: Nanomaterials (Basel). 2022 Jun 22;12(13):2137. doi: 10.3390/nano12132137 (PMC9268028; doi:10.3390/nano12132137)
Supplement: Supplementary file 1 [file nanomaterials-12-02137-s001.zip › nanomaterials-1758078-supplementary.pdf]

Supplementary information

Synthesis and Antibacterial Activity of Manganese-Ferrite/Silver Nanocomposite Combined with Two Essential Oils

Javiera Parada [1,2], Marcela Díaz [2], Edward Hermosilla [1,2], Joelis Vera [2,3], Gonzalo Tortella [1,2], Amedea B. Seabra [4], Andrés Quiroz [5], Emilio Hormazábal [5] and Olga Rubilar [1,2,*]

[1] Chemical Engineering Department, Universidad de La Frontera, Temuco P.O. Box 54-D, Chile; javiera.parada@ufrontera.cl (J.P.); edward.hermosilla@ufrontera.cl (E.H.); gonzalo.tortella@ufrontera.cl (G.T.)
[2] Biotechnological Research Center Applied to the Environment (CIBAMA-BIOREN), Universidad de La Frontera, Temuco P.O. Box 54-D, Chile; marcela.diaz@ufrontera.cl (M.D.); j.vera12@ufromail.cl (J.V.)
[3] Programa de Doctorado en Ciencias de la Ingeniería, Universidad de La Frontera, Temuco P.O. Box 54-D, Chile
[4] Center for Natural and Human Sciences, Federal University of ABC (UFABC), Santo André 09210-580, Brazil; amedea.seabra@ufabc.edu.br
[5] Departamento de Ciencias Químicas y Recursos Naturales, Universidad de La Frontera, Temuco P.O. Box 54-D, Chile; andres.quiroz@ufrontera.cl (A.Q.); emilio.hormazabal@ufrontera.cl (E.H.)
* Correspondence: olga.rubilar@ufrontera.cl

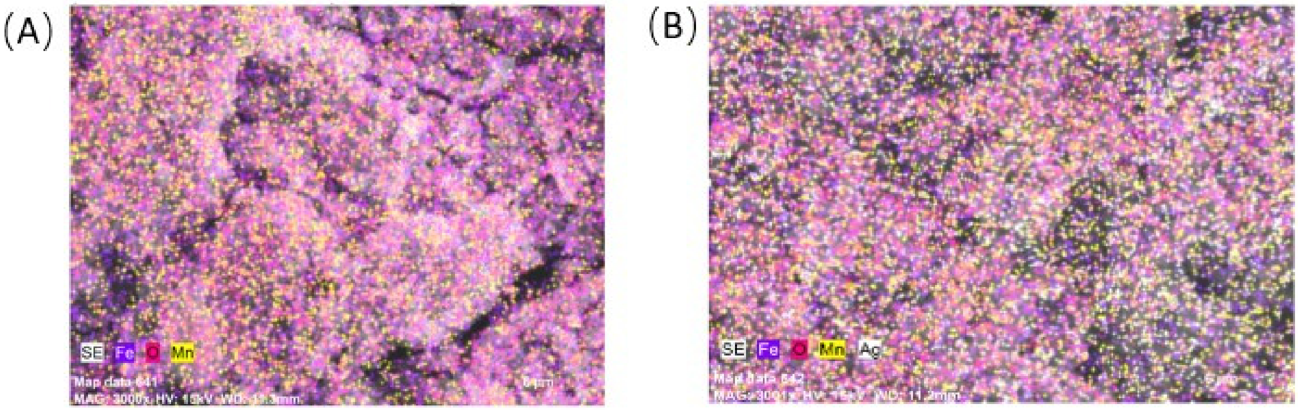

Figure S1. EDS elemental mapping for $MnFe_2O_4$ (A) and $MnFe_2O_4$/Ag-NC (B), respectively.
